# The oral cancer microbiome contains tumor space–specific and clinicopathology-specific bacteria

**DOI:** 10.3389/fcimb.2022.942328

**Published:** 2022-12-27

**Authors:** Bin Zeng, Jun Tan, Guangliang Guo, Zhengshi Li, Le Yang, Xiaomei Lao, Dikan Wang, Jingxin Ma, Sien Zhang, Guiqing Liao, Yujie Liang

**Affiliations:** ^1^ Department of Oral and Maxillofacial Surgery, Hospital of Stomatology, Guanghua School of Stomatology, Sun Yat-sen University, Guangzhou, Guangdong, China; ^2^ Guangdong Provincial Key Laboratory of Stomatology, Sun Yat-sen University, Guangzhou, Guangdong, China

**Keywords:** oral cancer (OC), oral microbiome, clinicopathology, intratumoral bacteria, 16S rDNA sequencing

## Abstract

The crosstalk between the oral microbiome and oral cancer has yet to be characterized. This study recruited 218 patients for clinicopathological data analysis. Multiple types of specimens were collected from 27 patients for 16S rRNA gene sequencing, including 26 saliva, 16 swabs from the surface of tumor tissues, 16 adjacent normal tissues, 22 tumor outer tissue, 22 tumor inner tissues, and 10 lymph nodes. Clinicopathological data showed that the pathogenic bacteria could be frequently detected in the oral cavity of oral cancer patients, which was positively related to diabetes, later T stage of the tumor, and the presence of cervical lymphatic metastasis. Sequencing data revealed that compared with adjacent normal tissues, the microbiome of outer tumor tissues had a greater alpha diversity, with a larger proportion of *Fusobacterium*, *Prevotella*, and *Porphyromonas*, while a smaller proportion of *Streptococcus*. The space-specific microbiome, comparing outer tumor tissues with inner tumor tissues, suggested minor differences in diversity. However, *Fusobacterium*, *Neisseria*, *Porphyromonas*, and *Alloprevotella* were more abundant in outer tumor tissues, while *Prevotella*, *Selenomonas*, and *Parvimonas* were enriched in inner tumor tissues. Clinicopathology-specific microbiome analysis found that the diversity was markedly different between negative and positive extranodal extensions, whereas the diversity between different T-stages and N-stages was slightly different. *Gemella* and *Bacillales* were enriched in T1/T2-stage patients and the non-lymphatic metastasis group, while *Spirochaetae* and *Flavobacteriia* were enriched in the extranodal extension negative group. Taken together, high-throughput DNA sequencing in combination with clinicopathological features facilitated us to characterize special patterns of oral tumor microbiome in different disease developmental stages.

## Introduction

The human microbiota confronts and symbioses with the human body and is therefore considered “the forgotten organ of the body” (O'Hara and Shanahan, 2006). Several body parts traditionally considered sterile, like the lower respiratory tract, have been identified uncultivable commensal microbial communities with the help of culture-independent sequencing approaches (Unger and Bogaert, 2017; Mishra et al., 2021). Disease states are also affected by microorganisms; more than 16% of cancers worldwide can be attributed to microbial infectious agents. Previous studies have found that carcinogenic bacteria aggregate in the tumor microenvironment and promote tumor development through direct stimulation or oncogenic agents. For example, *Fusobacterium nucleatum* in colorectal cancer and *Helicobacter pylori* in stomach cancer have been widely reported, and there is sufficient evidence outlining these bacteria’s critical role in cancer development (Kostic et al., 2012; Castellarin et al., 2012; Kostic et al., 2013; Rubinstein et al., 2013). However, regarding the crosstalk between tumors and microbiota, there still has many unsolved and unknown issues. In addition to carcinogenic bacteria *in situ*, recent studies on the tumor microbiome have demonstrated that commensal bacteria or distant bacteria also exhibit multiple effects on tumor biology and therapy (Jin et al., 2019; Riquelme et al., 2019; Lam et al., 2021; Pernigoni et al., 2021). Reportedly, commensal bacteria have the ability to promote lung cancer and resist anti-tumor treatment, while another study has shown that commensal bacteria facilitated the formation of anti-tumor macrophages (Jin et al., 2019; Lam et al., 2021). Distant bacteria from the gut can directly affect androgen deprivation therapy of prostate cancer by producing androgens or indirectly impact the outcome of pancreatic cancer by reshaping the tumor microbiome distantly (Riquelme et al., 2019; Pernigoni et al., 2021).

Compared with the studies on gut microbes, oral microbes are not studied in-depth, and bacteria’s role in oral cancer has not yet been revealed. Previous studies on oral tumor microbiome have mainly focused on comparing tumor tissues and normal or adjacent tissues (Zhang et al., 2019; Li et al., 2020; Sarkar et al., 2021; Torralba et al., 2020; Zhou et al., 2020) and found differential bacteria in the tumor microbiome. Nevertheless, the causal bacteria varied in different studies. The studies on tumor characteristics should not only be limited to comparing tumor or normal tissue but also need to consider more oncological factors, like TNM stage, pathological grade and types. Furthermore, intra-tumor heterogeneity of oral cancer can influence tumor prognosis. For example, spatial heterogeneity between the core and invasive front of the tumor or heterogeneity of tumor cell necrosis between apoptosis and necroptosis (Bankfalvi and Piffko, 2000; Li et al., 2020). Thus, characterization of the tumor microbiome remains essential to unraveling the role of microbes in oral cancer.

Our study analyzed the clinicopathological features of oral cancer patients and aimed to investigate the association between the presence of pathogenic bacteria and the oncological characteristics. Then we collected two types of tumor tissues (inner and outer), adjacent normal tissues, saliva, swabs, and lymph node tissues for 16S rRNA gene sequencing. We combined the high-throughput sequencing data with the findings of clinical data to explore whether the special pattern of the oral cancer microbiome was associated with the clinicopathological data.

## Materials and methods

### Study design

We conducted this study at the Hospital of Stomatology, Sun Yat-sen University, China. Eligible patients were at least 18 years of age, histologically confirmed oral squamous cell carcinoma, were treated with tumor resection and had complete clinical and pathological data in the e-medical system. Patients who had a recurrent tumor or had received radiotherapy or chemotherapy were excluded. In the 16S rRNA gene sequencing analysis, we excluded patients who had not undergone neck dissection or had insufficient tumor tissue for additional sequencing other than those used for pathological diagnosis.

The institutional ethics committee approved the study protocol and all the amendments of the Hospital of Stomatology, Sun Yat-sen University, China (Grant Number: ERC-[2018]-07) in accordance with the Declaration of Helsinki. All patients that underwent prospective specimen collection provided written informed consent before enrollment. Our study followed STROBE-metagenomics guidance and Strengthening The Organization and Reporting of Microbiome Studies (STORMS) to organize the research (Bharucha et al., 2020; Mirzayi et al., 2021).

### Data collection and statistical analysis

The clinicopathological data of the cohort were collected retrospectively, including basic information (i.e., age, gender, smoking status, alcohol consumption, history of antibiotic treatment, and systematic disease), tumor characteristics (i.e., site, appearance, TNM stage, pathological grade, extranodal extension), and inpatient test results (i.e., bacterial culture test and antimicrobial susceptibility test, blood test). Socioeconomic, behavioral, dietary, and biomedical characteristics were also collected. Data preprocessing (data verification, categorical variable conversion, and dummy variable conversion) was conducted prior to statistical analysis. The clinicopathological data were analyzed using the *χ* ^2^ test in SPSS (v26.0), and the two-tailed significance level of 0.05 was used. A statistician provided advice for the analysis.

A third-party inspection agency conducted the bacterial culture and antimicrobial susceptibility test (Guangzhou KingMed Diagnostics Group Co., Ltd.). Amies Agar Gel Transport Swabs (Thermo) were used and sent to the inspection agency at room temperature in two to three hours. Columbia blood agar plate and chocolate agar plate with vancomycin were used for aerobic culture. MacConkey agar plate and anaerobic blood agar plate were prepared for anaerobic culture. The plates were incubated for 24 to 48 hours at 37°C. Microbial identification was performed based on colony characteristics, Gram staining, and a series of standard biochemical reactions (Collee, 1996). Then the present/absent pattern of pathogenic bacteria was confirmed.

### Specimen collection

**Figure 1:**
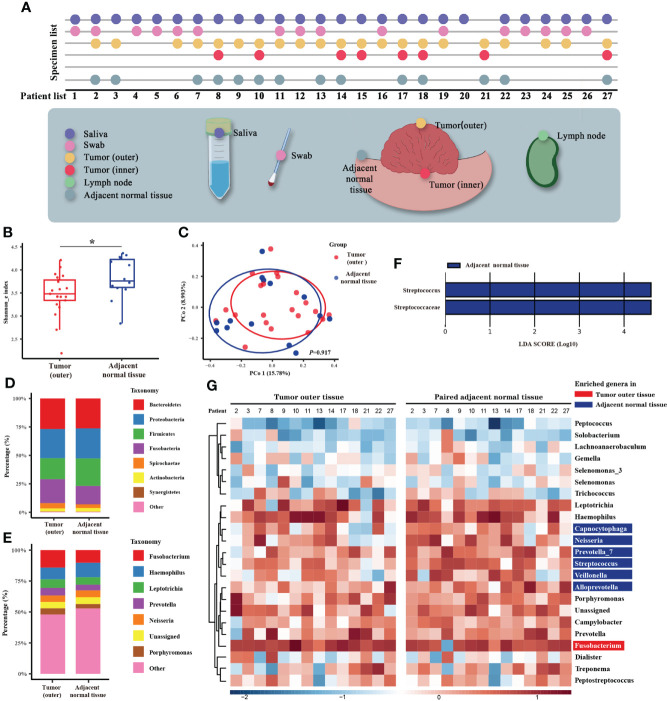
Microbiome analysis between tumor and normal adjacent tissues. **(A)** Diagram of the patient list and original collection sites of the specimens. Different colors mark six types of specimens, and detailed sampling sites are shown. In the specimen list, the dots represent the specimens that were qualified and sequenced. **(B)** Box plot of the alpha diversity of outer tumor tissues and normal adjacent tissues, shown by Shannon index. The horizontal bars within boxes represent medians. The tops and bottoms of boxes represent the 75th and 25th percentiles, respectively. **(C)** Principal coordinate analysis (PCoA) of outer tumor tissues and normal adjacent tissues based on Bray-Curtis distance. The dots represent the specimens, and the circles represent the microbial community associated with outer tumor tissues (red) and normal adjacent tissues (blue). **(D, E)** The microbial composition of the outer tumor tissues and normal adjacent tissues. D = phylum level and E = genera level. **(F)** Significantly different bacteria in two groups as determined by LEfSe analysis. **(G)** Alignment-based analysis based on the relative abundance of genera in the matched specimens of outer tumor tissues and normal adjacent tissues. Significantly different genera are marked with blue (for normal adjacent tissues) and red (outer tumor tissues).

Saliva and swab specimens were collected 2 hours after dinner on the day of admission. Patients were asked to rinse their mouths with saline in advance. After resting for 20 minutes, we collected 5 mL of saliva and wiped the surface of the tumor with a swab. Two swabs were collected, one for sequencing and one for bacterial culture. Two kinds of surface tissues (outer tumor tissue and adjacent normal tissue) and inner tumor tissues were collected by the surgeon immediately after tumor resection (**Figure 1A**). Outer tumor tissue was collected at a depth of less than 2mm on the surface of the tumor, which was considered that there might be a potential microbial transformation with the oral cavity. Adjacent normal tissue was collected at the negative margin of tumor resection. Inner tumor tissue was collected at the front of the invasion of the tumor, which was considered none of the microbial transformations with the oral cavity. Lymph nodes suspected of metastasis through preoperative MRI were separated from neck dissection tissue. Sterile instruments were used for sampling, and the instruments were recleaned with 75% ethanol and sterile gauze every time before collecting another sample to avoid contamination. Then the specimens were temporarily stored in liquid nitrogen. After confirmation of the pathological status by a pathologist, the specimens were transferred to the lab and stored at -80°C.

### Oral cancer microbiome characterization using 16S rRNA gene amplification and sequencing

Briefly, DNA extraction, PCR amplification of 16S ribosomal RNA (rRNA) gene, and sequencing were performed at The Beijing Genomics Institute according to our protocol. It was adapted from the methods developed for the NIH-Human Microbiome Project (HMP) and Earth Microbiome Project (EMP). In detail, at least two sections of 0.5 cm^3^ of cryogenic oral cancer tissue were aseptically sent for sequencing, and paired normal oral tissues were used as controls. Genomic DNA from these samples was extracted using the FastDNA SPIN Kit (MP Biomedicals). DNAs were measured by Agilent 2100 Bioanalyzer (Agilent Technologies, Inc) and subsequently diluted to 3.5 ng µl^–1^. The 16S rDNA V4 region was adopted for HMP and EMP; the amplified data included maximized data resolution. The following bacterial primers for the V4 region of the 16S rRNA region were used in combination: 515F (5’-GTGCCAGCMGCCGCGGTAA-3’) and 806R (5’-GGACTACHVGGGTWTCTAAT-3’). The blank wells were used as the negative control. Each sample was amplified in triplicate in a 30 μl reaction system, containing 3 µl of diluted DNA, 0.75 U PrimeSTAR HS DNA polymerase, 1x PrimSTAR buffer (Takara), 0.2 mM deoxyribonucleoside triphosphates (dNTPs) and 10 pM of barcoded forward and reverse primers. After an initial denaturation step at 98 °C for 30 s, the targeted region was amplified by 25 cycles of 98 °C for 10 s, 55 °C for 15 s, and 72°C for 60 s, followed by a final elongation step of 5 min at 72°C. The PCR products were measured by Nanodrop (NanoDrop 2000C, Thermo Scientific) and diluted to 10 ng µl^–1^ as templates for the second step of the PCR. All samples were amplified in triplicate with second-step primers with eight cycles. Before high-throughput sequencing, PCR product electrophoresis experiments carried out a quality inspection of samples to exclude low-quality specimens. Final amplicon libraries were purified twice using an Agencourt AMPure XP Kit (Beckman Coulter) and subjected to a single sequencing run on the HiSeq 2500 platform (Illumina Inc).

### Bioinformatics analysis on 16S rRNA gene sequencing

The paired reads were processed using USEARCH (v.10.0) (Edgar, 2010), R (v.3.6.0), and in-house scripts (Zhang et al., 2019). The paired reads were processed in the following steps by USEARCH: joining of paired-end reads and relabeling of sequencing names, removal of barcodes and primers, filtering of low-quality reads, dereplication and cluster into operational taxonomic units (OTUs) with 97.0% similarity. Then OTUs were aligned to the SILVA Database v128 (Quast et al., 2013; Edgar et al., 2011), and removed sequences from the chimera and host plastids. Human DNA and other contaminants were eliminated. The OTU table was generated by USEARCH. The alpha and beta diversity were computed using USEARCH. The Shannon index was used to measure the alpha diversity between groups. Bray-Curtis dissimilarity was used to measure the beta diversity across specimens, and the data were visualized *via* principal coordinate analysis. The taxonomy of the representative sequences was classified with the SILVA Database v128 (Quast et al., 2013). Linear discriminant effect size (LEfSe) was used for linear discriminant analysis to screen for specific genera (Segata et al., 2011) with an LDA score greater than 2. We adopted the alignment-based analysis according to a recent study (Castellarin et al., 2012). The alignment-based analysis only obtained the predominant genera found in both tumor and normal specimens. Rare genera and those specific to tumor only or adjacent normal specimens only were considered sampling bias in the alignment-based analysis. The different genus was considered to have at least a two-fold variation in relative abundance between most matched specimens in the two groups. Functional and phenotype prediction was finished by PICRUSt (Langille et al., 2013) and BugBase (Ward et al., 2017). The figures were visualized using the ggplot2 v.2.2.1 package in R (v.3.6.0) (Wilkinson, 2011).

## Results

The entire cohort for clinicopathological analysis included 218 patients enrolled between Aug 2016 and Nov 2020 (**Supplementary Figure 1**). From these patients, six types of specimens (26 saliva, 16 swabs from the surface of tumor tissues, 16 adjacent normal tissues, 22 tumor outer tissues, 11 tumor inner tissues, and 10 lymph nodes) were collected from 27 serial patients between Feb 2020 and Nov 2020 for 16S rRNA gene sequencing (**Supplementary Figure 1**). After quality assessment, a total of 26 saliva, 16 swabs, 14 adjacent normal tissues, 21 tumor outer tissues, and 8 tumor inner tissues were eligible. The sequencing procedure was completed, and the raw data was returned for bioinformatics analysis (**Figure 1A**).

### Statistical analysis of clinicopathological data

The entire cohort of 218 oral cancer patients included 149 (68.3%) male and 69 (31.7%) female patients with an average age of 52.4 years. Demographic and tumor characteristics are presented in **Table 1**. Most patients were pathologically diagnosed with squamous cell carcinoma of the tongue (108, 49.5%) or buccal (41, 18.8%), or gingival (32, 14.7%) cancer. 89 (40.8%) patients were pathologically confirmed as T1/T2 stage, and 57 (26.1%) at T3 stage, 72 (33.0%) at T4 stage. Nearly half of the patients (112, 51.4%) were diagnosed with cervical lymph node metastasis. 105 (48.2%) of the 218 patients were found to have pathogenic bacteria in bacterial culture tests. We analyzed the association between clinicopathological data and bacterial culture test results using *χ* ^2^ test. And we found that the presence of pathogenic bacteria has positive relationships with diabetes (*P*=0.011), higher T stage of the tumor (*P*<0.001), and the presence of cervical lymphatic metastasis (*P*=0.040).

**Table 1:** Patient demographic and clinicopathological characteristics of the entire cohort of 218 patients.

			Bacterial culture test		
			Negative (N = 113)	Positive (N = 105)	*P*
Basic information	Age		52.06 ± 12.59	52.81 ± 12.44	0.660
	BMI index		22.90 ± 2.68	22.71 ± 2.92	0.610
	Gender	Male	75 (66.4%)	74 (70.5%)	0.613
		Female	38 (33.6%)	31 (29.5%)	
	Smoking	Yes	50 (44.2%)	56 (53.3%)	0.228
		No	63 (55.8%)	49 (46.7%)	
	Drinking	Yes	19 (16.8%)	22 (21.0%)	0.543
		No	94 (83.2%)	83 (79.0%)	
	History of antibiotic treatment	Yes	24 (21.2%)	27 (25.7%)	0.535
		No	89 (78.8%)	78 (74.3%)	
	Diabetes	Yes	9 (8.0%)	22 (21.0%)	0.011*
		No	104 (92.0%)	83 (79.0%)	
Tumor features	Site				0.143
		Tongue	65 (57.5%)	43 (41.0%)	
		Buccal	21 (18.6%)	20 (19.0%)	
		Gingival	13 (11.5%)	19 (18.1%)	
		Palate	4 (3.5%)	5 (4.8%)	
		Oral floor	8 (7.1%)	12 (11.4%)	
		Others	2 (1.8%)	6 (5.7%)	
	Appearance classification				0.854
		Exogenous	54 (47.8%)	52 (49.5%)	
		Ulcer	48 (42.5%)	45 (42.9%)	
		Infiltrating	11 (9.7%)	8 (7.6%)	
	Pathological T staging				<0.001*
		T1/T2	56 (49.6%)	33 (31.4%)	
		T3	37 (32.7%)	20 (19.0%)	
		T4	20 (17.7%)	52 (49.5%)	
	Pathological N staging				0.058
		N0	63 (55.8%)	43 (41.0%)	
		N1	25 (22.1%)	22 (21.0%)	
		N2	22 (19.5%)	37 (35.2%)	
		N3	3 (2.7%)	3 (2.9%)	
	Cervical lymph node metastasis	Yes	50 (44.2%)	62 (59.0%)	0.040*
		No	63 (55.8%)	43 (41.0%)	
	Pathological grading				0.064
		High	83 (73.5%)	62 (59.0%)	
		Moderate	29 (25.7%)	40 (38.1%)	
		Poor	1 (0.9%)	3 (2.9%)	
Inpatient test result	White blood cell (10^9^/L)		6.50 ± 1.74	6.82 ± 2.35	0.254
	Neutrophils (10^9^/L)		3.84 ± 1.47	4.21 ± 2.09	0.132
	Lymphocyte (10^9^/L)		2.04 ± 0.76	1.92 ± 0.65	0.217
	Neutrophils-lymphocyte ratio		2.12 ± 1.15	2.43 ± 1.53	0.091
	Monocytes (10^9^/L)		0.44 ± 0.14	0.62 ± 1.15	0.094
	C-reactive protein (mg/L)		2.76 ± 5.92	2.85 ± 4.61	0.903
	Procalcitonin (ng/mL)		0.06 ± 0.02	0.28 ± 2.18	0.304
	Albumin (g/L)		40.92 ± 3.35	41.33 ± 3.82	0.404

Bold value with * indicates p value < 0.05.

### Tumor-specific microbiome of oral cancer

The characteristics of 27 patients whose samples were sent for microbial sequencing are listed in **Supplementary Table 1**. Raw 16S rRNA gene sequencing data generated a total of 1,471 OTUs (**Supplementary Table 2**). Metadata is available in **Supplementary Table 3**. We first compared the microbiomes of outer tumor tissues and adjacent normal tissues. As measured by the Shannon index, decreased alpha diversity of the oral tumor microbiome was observed in outer tumor tissues (**Figure 1B**, *P* = 0.028). However, no significant difference in beta diversity was observed between these two groups (**Figure 1C**, *P* = 0.917). The taxonomy bar plot showed a greater proportion of *Fusobacterium*, *Prevotella*, and *Porphyromonas* in the outer tumor tissues (**Figures 1D, E**). LEfSe analysis found that a distinct genus, *Streptococcus*, was significantly decreased in outer tumor tissues (**Figure 1F**).

Interestingly, when all the specimens (saliva, swab, outer tumor tissue, inner tumor tissue, and adjacent normal tissue) were clustered according to beta diversity, it was noted that specimens from the same patient tended to be clustered together. In contrast, specimen type or tumor characteristics only exerted a minor effect on the oral tumor microbiome (**Supplementary Figures 2**, **3**). Based on this finding, our further analysis not only relied on standard microbiome analysis methods but also used alignment-based methods to compare paired specimens from the same person (Castellarin et al., 2012). We obtained markedly disproportionate alignments between the two groups and noted *Fusobacterium* enriched in the outer tumor tissues, whereas *Capnocytophaga*, *Neisseria*, *Prevotella*, *Streptococcus*, *Veillonella*, and *Alloprevotella* enriched in the normal adjacent tissues (**Figure 1G**).

### Space-specific microbiome of oral cancer

**Figure 2:**
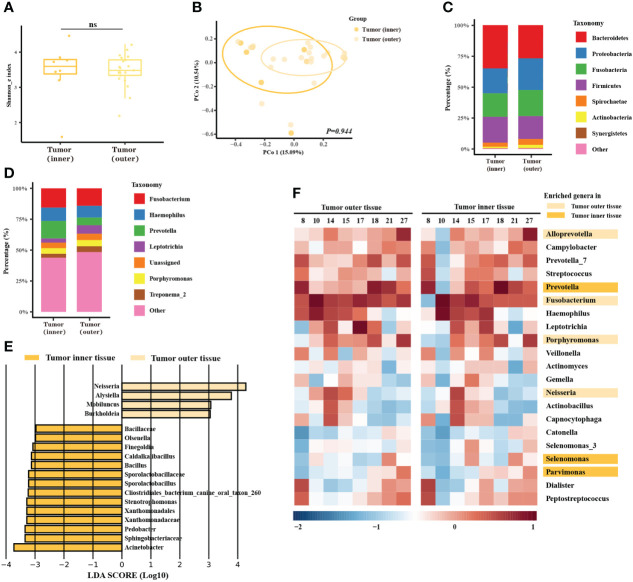
Microbiome analysis between different spatial features of the tumors. **(A)** Box plot of the alpha diversity of outer and inner tumor tissues, shown by Shannon index. The horizontal bars within boxes represent medians. The tops and bottoms of boxes represent the 75th and 25th percentiles, respectively. **(B)** Principal coordinate analysis (PCoA) of outer tumor tissues and normal adjacent tissues based on Bray-Curtis distance. The dots represent the specimens, and the circles represent the microbial community associated with the outer tumor tissues (light orange) and normal adjacent tissues (dark orange). **(C, D)** The microbial composition of the outer tumor tissues and inner tissues. C = phylum level and D = genera level. **(E)** Significantly different bacteria in two groups as determined by LEfSe analysis. **(F)** Alignment-based analysis based on the relative abundance of genera in the matched specimens of outer and inner tumor tissues. Significantly different genera are marked with dark orange (inner tumor tissues) and light orange (outer tumor tissues).

As previous studies had found that the characteristics of the invasive tumor front were associated with prognosis, our next analysis section concerned the microbial differences between the outer and inner tumor tissues. The microbial community of the outer and inner tissues was only slightly different in alpha diversity (**Figure 2A**, *P* = 0.873) or beta diversity (**Figure 2B**, *P* = 0.944). The taxonomy bar plot showed a greater proportion of *Prevotella* in the inner tumor tissue (**Figure 2C, D**), consistent with later alignment-based analysis. LEfSe analysis showed several different genera. The abundance of *Neisseria* was enriched in the microbial community of the outer tumor tissue versus the inner tissue, but most of enriched genera were rare-abundance (**Figure 2E**). In the alignment-based analysis, *Fusobacterium*, *Neisseria*, *Porphyromonas*, and *Alloprevotella* were more abundant in the outer tumor tissues, while *Prevotella*, *Selenomonas*, and *Parvimonas* were over-abundant in the inner tumor tissues (**Figure 2F**).

Moreover, functional prediction showed that functional pathways related to metabolism, genetic information processing, environmental information processing, and human disease were differentially enriched in the oral tumor microbiome of inner tissue at KEGG level 1(**Supplementary Figure 4A**). At KEGG level 3, ribosome, pyrimidine metabolism, purine metabolism, peptidases, DNA repair, recombination proteins, and ABC transporters were enriched in the oral tumor microbiome of inner tumor tissue (**Supplementary Figure 4B**). Phenotype prediction showed that the microbiome of inner tumor tissue was more likely to be anaerobic, to contain more mobile elements, and less form biofilm (**Supplementary Figures 4C–F**).

### Clinicopathology-specific microbiome of oral cancer

**Figure 3:**
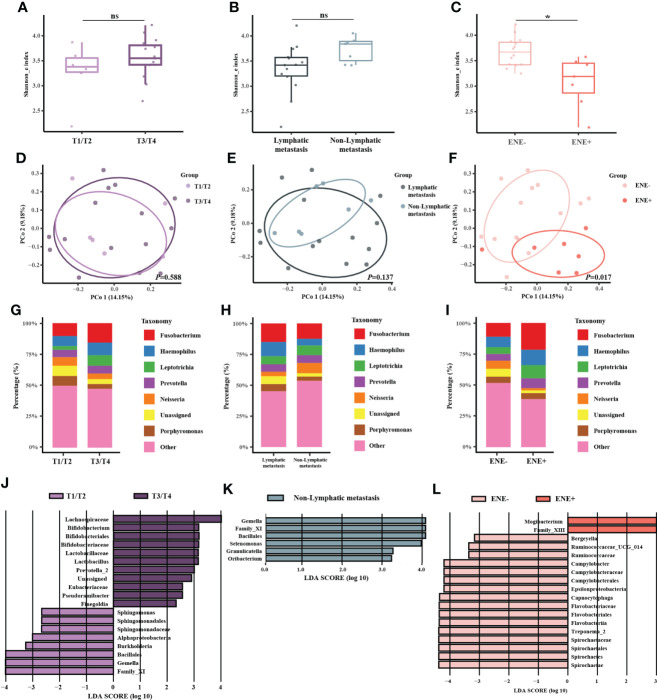
Microbiome analysis between different T-stages, N-stages, and ENE status. **(A–C)** Box plot of the alpha diversity of outer tumor tissues between T1/T2-stage and T3/T4-stage, between lymphatic metastasis and non-lymphatic metastasis, and between ENE- and ENE+ shown by Shannon index. The horizontal bars within boxes represent medians. The tops and bottoms of boxes represent the 75th and 25th percentiles, respectively. **(D–F)** Principal coordinate analysis (PCoA) of outer tumor tissues and normal adjacent tissues based on Bray-Curtis distance. The dots represent the specimens, and the circles represent the microbial community associated with T1/T2-stage (light purple) and T3/T4-stage (dark purple) in **(D)**, lymphatic metastasis (dark green) and non-lymphatic metastasis (light green) in **(E)**, ENE-(light pink) and ENE+ (pink) in **(F–I)** The microbial composition of different groups at the genera level. **(J–L)** Significantly different bacteria in the two groups as determined by LEfSe analysis.

Next, we associated the clinicopathological data with the oral microbiome based on the outer tumor tissue sequencing data. Alpha diversity was higher in the ENE negative (ENE-) group (**Figure 3C**, *P* = 0.003), while the other two comparisons (T1/T2-stages group versus T3/T4-stages group, lymphatic metastasis group versus non-lymphatic metastasis group) were not significantly different (**Figure 3A**, *P* = 0.217; **Figure 3B**, *P* = 0.068). Beta diversity did not show any significantly different between different T-stages or N-stages group (**Figure 3D**, *P* = 0.588; **Figure 3E**, *P* = 0.137), but it was markedly different between different states of ENE (**Figure 3F**, *P* = 0.017). The taxonomic bar plot showed that the proportions of *Fusobacterium* and *Haemophilus* were enriched in the T3/T4-stages, lymphatic metastasis, and the ENE+ group, while the proportion of *Neisseria* was more abundant in the T1/T2-stages, non-lymphatic metastasis, and the ENE- group (**Figures 3G-I**). Moreover, the genera *Porphyromonas* exhibited contradictory trends: the proportion was higher in the T1/T2 stages but also the lymphatic metastasis group. In the LEfSe analysis, we found that *Gemella* and *Bacillales* were significantly abundant in the T1/T2-stages and the non-lymphatic metastasis group (**Figures 3J, K**), while *Spirochaetae* and *Flavobacteria* were enriched in the ENE- group (**Figure 3L**).

## Discussion

Our work was inspired by the findings of the commonly used bacterial examination in clinical practice, which indicated that most oral cancer patients had dysbiotic microbiome and pathogenic bacteria. Moreover, this phenomenon was correlated with the oncological features of patients, including T staging and cervical lymphatic metastasis. Therefore, we performed high-throughput sequencing to unveil the special patterns of the oral tumor microbiomes in different oncological states.

The bacterial culture and antimicrobial susceptibility test were of diagnostic value for determining whether there was a bacterial infection at the body sites, such as the thoracic cavity, abdominal cavity, and joints, in which normal puncture fluid should be sterile. However, the oral cavity was a non-sterile environment where more than 280 bacterial species had been isolated in culture and formally named (Dewhirst et al., 2010). Based on the culturomics techniques, we found pathogenic bacteria had been detected in most oral cancer patients. These findings alerted clinicians to routine peri/post-operative antibiotic prophylaxis in oral cancer patients. The American Society of Health-system Pharmacists’ antimicrobial prophylaxis guidelines recommended prophylaxis with agents, such as cefazolin plus metronidazole, for clean-contaminated wounds after oral cancer surgery (Veve et al., 2017). However, according to the high detection rate of pathogenic bacteria in oral cancer patients, we recommended the routine bacterial culture and antimicrobial susceptibility test before surgery and the use of antibiotics based on the results. Furthermore, it drew our attention to perturbations of tumor microbiome in oral cancer patients.

Cultivation-independent methods, like 16S rRNA gene sequencing, have widely replaced cultivation methods to discover the microbiome. In this study, we compared the oral microbiome between the tumor and adjacent normal tissue using 16S rRNA gene sequencing. Consistent with several studies, we found *Streptococcus* more abundant in adjacent normal tissues (Zhang et al., 2019; Torralba et al., 2020). LEfSe was commonly applied in microbiome analysis. However, different bacteria identified by LEfSe in studies were mainly rare genera, meaning that minor fluctuations or sampling bias could be reflected as significant differences (Zhou et al., 2020; Sarkar et al., 2021). In addition, previous studies collected specimens of multiple origins (saliva, swab, tissue) and found no significant differences between these specimens if grouped by specimen type. However, the investigators did not cluster the different specimen types and concluded no change in bacteria abundance (Zhang et al., 2019; Torralba et al., 2020; Li et al., 2020). In our study, cluster analysis addressed this issue. We included a wide range of oral specimens, including saliva, swabs, outer tumor tissues, inner tumor tissues, and adjacent normal tissues, and found that the microbiome was primarily clustered by individuals. In contrast, the tumor site or other factors could exert a minor influence on the oral tumor microbiome.

Another interesting finding in our study was the difference between inner and outer tumor tissues. Although it is known that the tumor invasion front is of great value for evaluating tumor prognosis, determining differences between the microbes in the inner and outer surface of the tumor has not yet been explored. A recent study applied RNA fluorescence *in situ* hybridization with bacterial 16S rRNA probes on whole-section slides of multiple solid tumors (Nejman et al., 2020). It reported that the whole layer of lung tumors, breast tumors, and melanoma had been observed the presence of microorganisms. However, the author used the entire tumor bulk as one sample for subsequent bacterial culture or sequencing and did not distinguish between the surface and deeper sections. Our study collected inner tumor tissue at the forefront of invasion and ensured clear tissue separation between the surface and the deeper section. The sequencing analysis found that the proportion of *Prevotella*, *Selenomonas*, and *Parvimonas* was enriched in the inner tumor tissues. These bacteria mostly grow in an anaerobic environment and are associated with biofilms in the subgingival dental plaque. Nevertheless, due to the limitations of 16S rRNA sequencing, we could not determine which species or strains were specific to the inner part of tumor tissues. For a more mechanistic understanding of the complex microbial community in oral cancer tissue, microbiome-associated studies should go beyond spatial investigation and be put into both temporal and spatial contexts, where the adapted concepts and methodological approaches need to be updated accordingly (Raes and Bork, 2008; Fischbach, 2018; Wang et al., 2020; Zheng et al., 2022; Sun et al., 2022).

We also collected the patients’ clinicopathological data and correlated these with the oral microbiome. Generally, a higher T-stage tended to be accompanied by a higher N-stage. Our results showed that *Fusobacterium* might be oncogenic bacteria, as it was found in a higher proportion in the T3/T4-stages, lymphatic metastasis, and the ENE+ group. However, some results of the microbiome analysis were contradictory. For example, there was a higher proportion of *Porphyromonas* in the T1/T2-stages and lymphatic metastasis groups. Due to the limitations of our associated study, we could not elucidate and verify the causal mechanisms of this contradiction (Cummins and Tangney, 2013). In other reports, TNM stage was applied in the oral saliva microbiome analysis (Yang et al., 2018; Takahashi et al., 2019; Chen et al., 2020), but only one study applied this clinical characteristic in tumor tissue microbiome analysis (Yang et al., 2021). Consistent with our results, *Fusobacterium* was highly abundant in the OSCC patients. But there was still a lack of other clinicopathological groups in tissue microbiome analysis. In addition to the clinicopathological data, other genetic or microstructural characteristics, such as expansive or infiltrative patterns at the invasion front, the composition of tumor-infiltrating lymphocytes, and the degree of tumor angiogenesis, also require further exploration.

This study had some limitations. (1) We found that the amount of DNA extracted from the lymph node tissues was not sufficient to perform 16S rRNA gene sequencing successfully. However, a previous study sequenced 14 lymph node specimens and found a microbial similarity between lymph nodes and primary tumors (Shin et al., 2017). Another culturomics study found that nearly half of lymph nodes from oral cancer patients after neck dissection were cultivable (Sakamoto et al., 1999). The cultured bacteria contained mainly oral bacteria but also had some species (e.g., *Escherichia*, *Staphylococcus*) that were potentially from the gut, skin, or contamination. Our future studies will focus on the characteristics of the microbiome in metastatic lymph nodes and elucidate the possible mechanisms in promoting or preventing cervical lymphatic metastasis. (2) The technical measures to minimize and control potential contamination were still insufficient (Eisenhofer et al., 2019; Nejman et al., 2020). Although no controls during the sampling procedure were set, we did set DNA extraction controls, no-template PCR amplification controls, and sequencing run controls. (3) The 16S sequencing data were clustered into OTU with a 97% similarity in this study. We did not perform the analysis of OTUs at a higher similarity, like 99% or 100%, which might allow us to resolve the fine-scale variation in oral microbiome that can result in a higher prediction accuracy for classifying host phenotypes than that reported in this study (Callahan et al., 2016). (4) Low microbial biomass samples might contain DNA levels similar to blank controls, including air, the built environment, and blood (Eisenhofer et al., 2019). The human tissue samples also had an ultra-lower input of microbial biomass, often leading to the detection of non-biologically relevant taxa and resulting in controversial results across studies. Hence, we recommended the alignment-based analysis where we can analyze tissue microbiomes with spatially-paired samples and negative controls (Castellarin et al., 2012). It would be a better approach to avoid potentially introducing extremely rare genera and highly unique compositions to final biological conclusions. Furthermore, more studies are needed to perform the rational validation with in-slide examinations like immunohistochemistry using lipopolysaccharide or lipoteichoic acid or fluorescence *in situ* hybridization using 16S rDNA probes.

In summary, this study characterized the oral tumor microbiome associated with different specimens, such as inner tumor tissues and outer tissues, and disease state variables, such as T-stages, N-stages, and ENE states. Also, we identified a previously unexplored microbial pattern that revealed the space-specific microbial composition at the invasive front of the tumor. These findings provide insights for future research exploring microbiome-centric mechanisms of oral cancer carcinogenesis.

## Data availability statement

The datasets presented in this study can be found in online repositories. The names of the repository/repositories and accession number(s) can be found below: NCBI BioProject - PRJNA813634.

## Ethics statement

The studies involving human participants were reviewed and approved by Hospital of Stomatology, Sun Yat-sen University, China. The patients/participants provided their written informed consent to participate in this study.

## Author contributions

BZ, XML, GQL, and YJL conceived and contributed to the study concept and design. XML, SEZ, and YJL recruited the participants. BZ, ZSL, and JT collected the specimens and clinicopathological data, and GLG, DKW and JXM provided partial help during the collection. GLG, and LY developed the statistical analysis workflows. BZ and JT developed the 16S rRNA gene profiling workflows. BZ, GLG, JT, and ZSL completed all analyses and finished the manuscript. GQL and YJL supervised the study. All authors contributed to the article and approved the submitted version.

## Funding

This work was supported by the National Natural Science Foundation of China (No. 81972544; No. 82072995).

## Acknowledgments

We thank all participants in this study and the support from all colleagues in the Department of Oral and Maxillofacial Surgery, Hospital of Stomatology, Sun Yat-sen University. We thank LetPub (www.letpub.com) for its linguistic assistance during the preparation of this manuscript.

## Conflict of interest

The authors declare that the research was conducted without any commercial or financial relationships that could be construed as a potential conflict of interest.

## Publisher’s note

All claims expressed in this article are solely those of the authors and do not necessarily represent those of their affiliated organizations, or those of the publisher, the editors and the reviewers. Any product that may be evaluated in this article, or claim that may be made by its manufacturer, is not guaranteed or endorsed by the publisher.

## Supplementary material

Supplementary Figure 1 The study flowchart.

Supplementary Figure 2 The hierarchical clustering analysis of 85 specimens based on beta diversity. Cain represents inner tumor tissues, caout outer represents tumor tissues, para represents normal adjacent tissues, swab and saliva represent swab and saliva. Numbers are from the patient numbers shown in .

Supplementary Figure 3 Principal coordinate analysis (PCoA) of 85 specimens based on Bray-Curtis distance. The dots represent the specimens, and the circles represent the microbial community associated with inner tumor tissues (red), outer tumor tissues (yellow), adjacent normal tissues (grey), saliva (blue), and swabs (pink).

Supplementary Figure 4
**(A, B)** Bar plot of the predicted function of inner tumor tissues and outer tumor tissues at KEGG level 1 **(A)** and KEGG level 3 **(B)** by PICRUSt. **(C–F)** Scatter plot of predicted phenotypes of inner and outer tumor tissues by BugBase. Four kinds of phenotypes were shown: anaerobic **(C)**, aerobic **(D)**, mobility **(E)**, and biofilm-forming **(F)**.

Supplementary Table 1 Patient demographic and clinicopathological characteristics of the 27 patients in the microbiome analysis.

Supplementary Table 2 The table of OTUs for 27 patients and 85 specimens.

Supplementary Table 3 The metadata of the 27 patients in the microbiome analysis.

## Abbreviations

OUT, Operational taxonomic units; ENE, Extranodal extension; LEfSe, Linear discriminant effect size; OSCC, Oral squamous cell carcinoma; STORMS, Strengthening The Organization and Reporting of Microbiome Studies; 16S Rrna, 16S ribosomal RNA.
