# Supplementary figures and images for: The oral cancer microbiome contains tumor space–specific and clinicopathology-specific bacteria
Source: Front Cell Infect Microbiol. 2022 Dec 27;12:942328. doi: 10.3389/fcimb.2022.942328 (PMC9831678; doi:10.3389/fcimb.2022.942328)

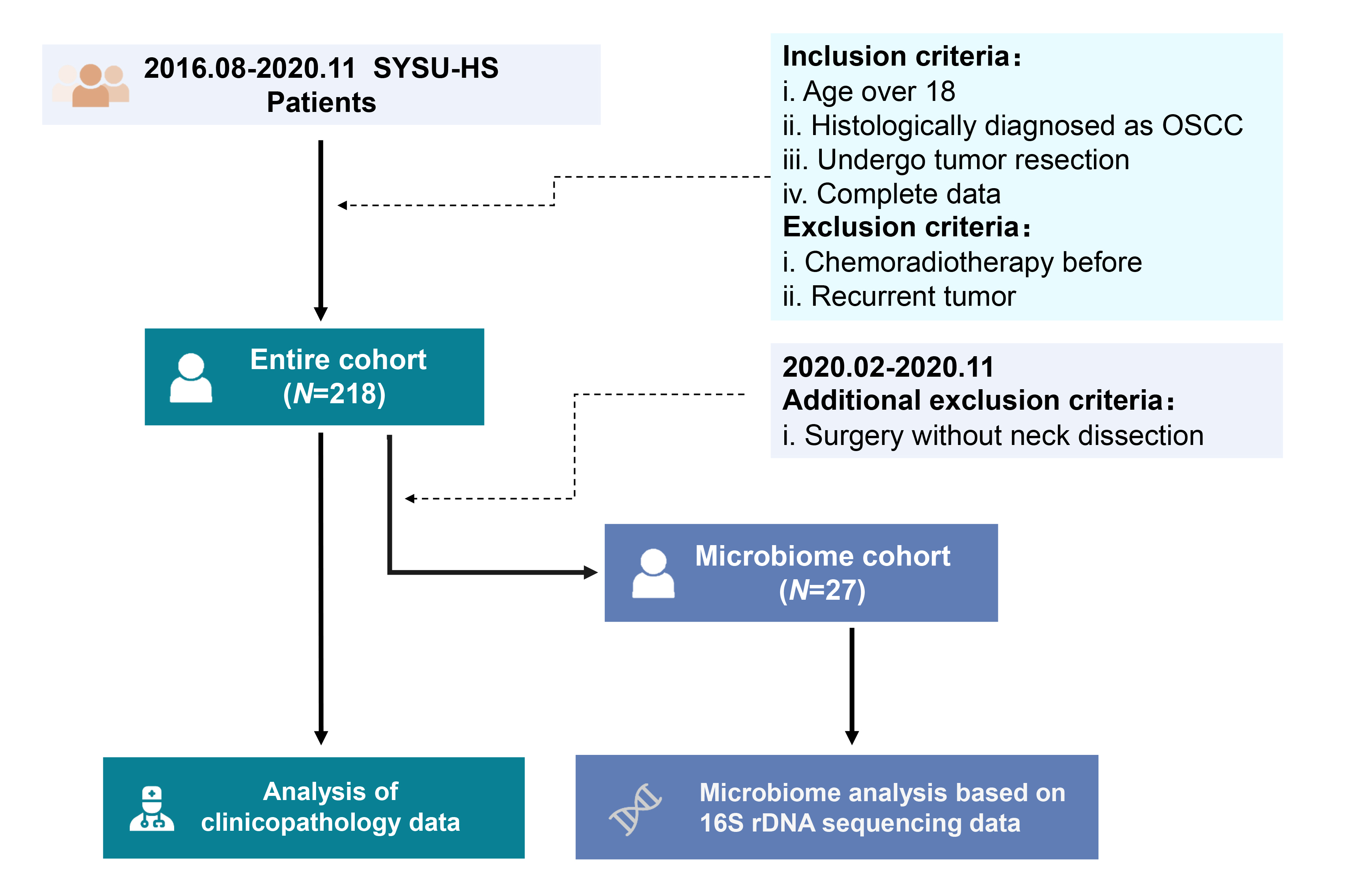

Supplement: Supplementary Figure 1 — The study flowchart. [file Image_1.jpeg]

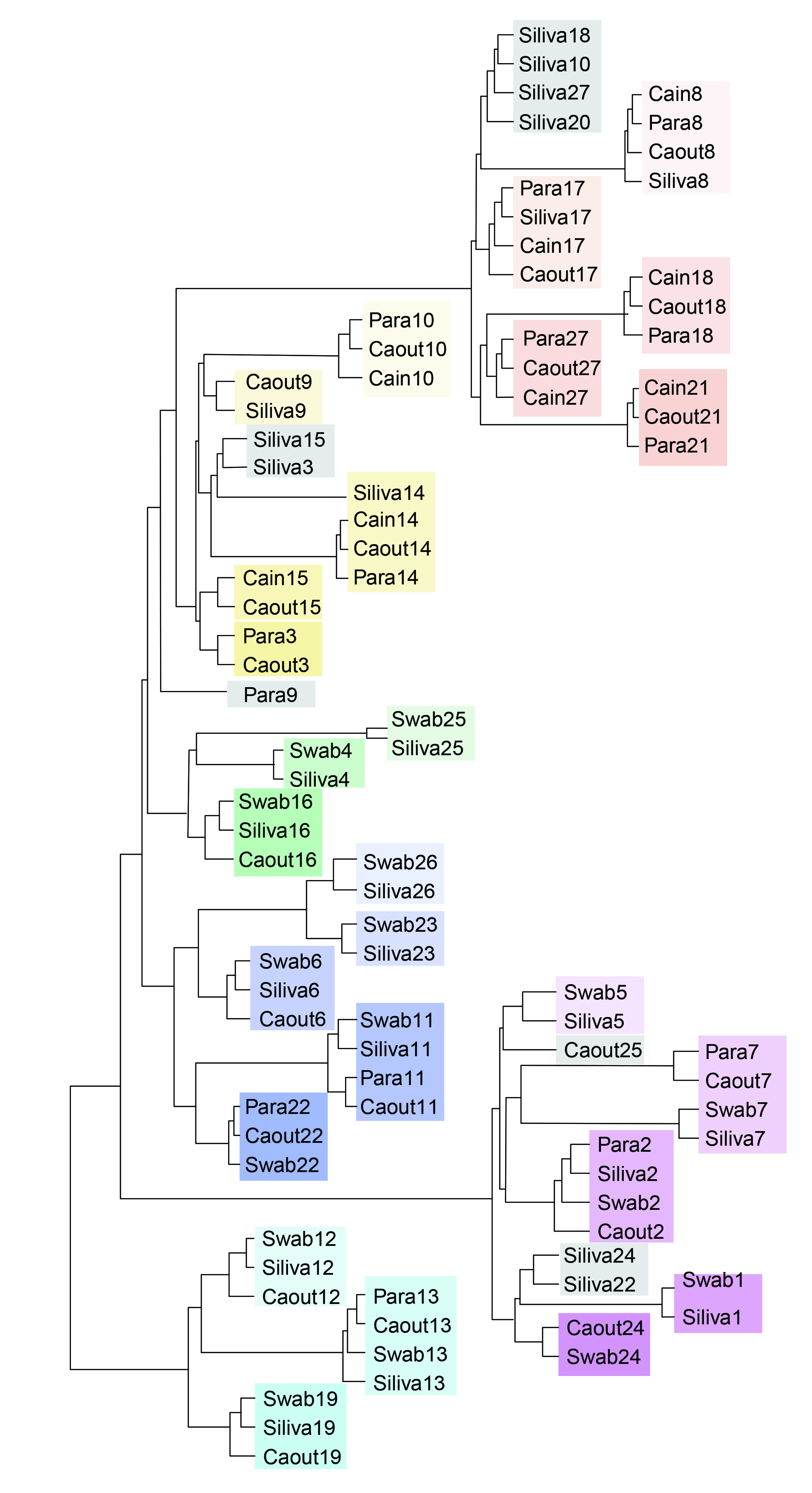

Supplement: Supplementary Figure 2 — The hierarchical clustering analysis of 85 specimens based on beta diversity. Cain represents inner tumor tissues, caout outer represents tumor tissues, para represents normal adjacent tissues, swab and saliva represent swab and saliva. Numbers are from the patient numbers shown in . [file Image_2.jpeg]

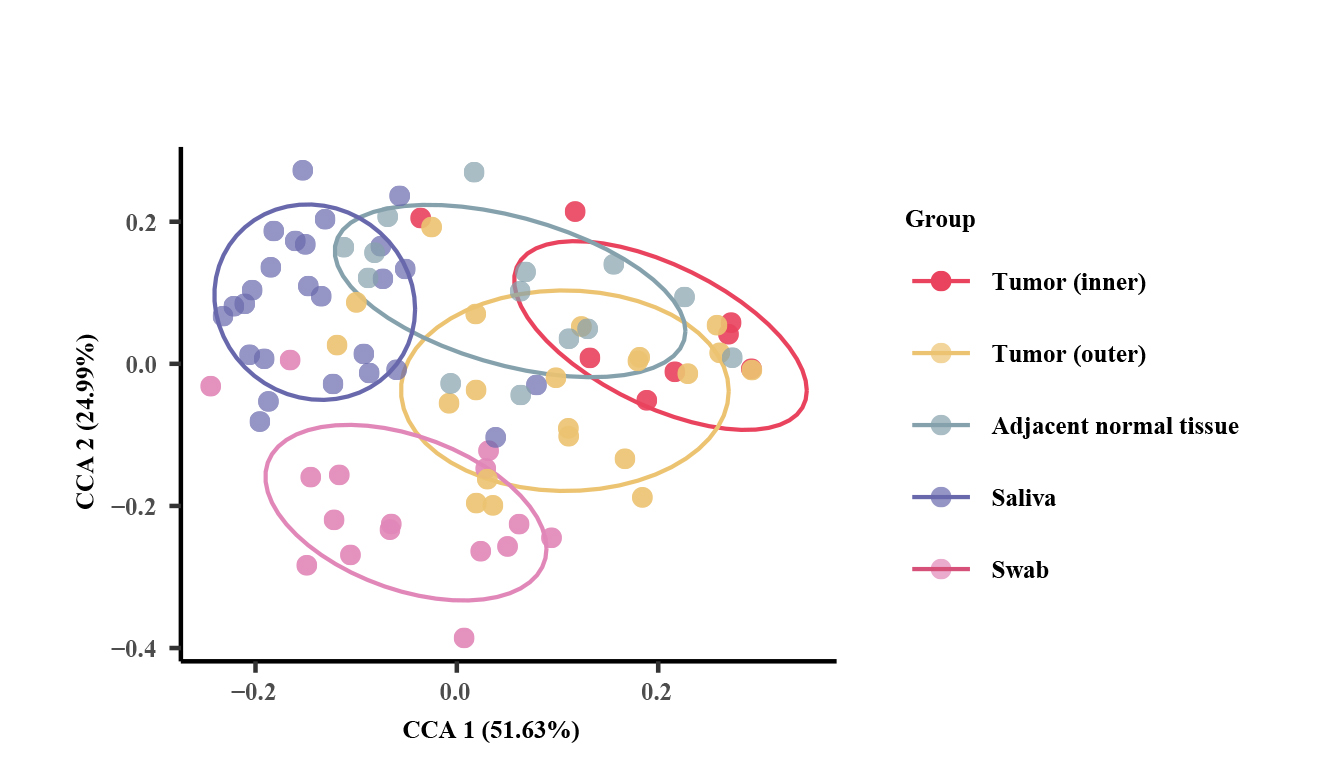

Supplement: Supplementary Figure 3 — Principal coordinate analysis (PCoA) of 85 specimens based on Bray-Curtis distance. The dots represent the specimens, and the circles represent the microbial community associated with inner tumor tissues (red), outer tumor tissues (yellow), adjacent normal tissues (grey), saliva (blue), and swabs (pink). [file Image_3.jpeg]

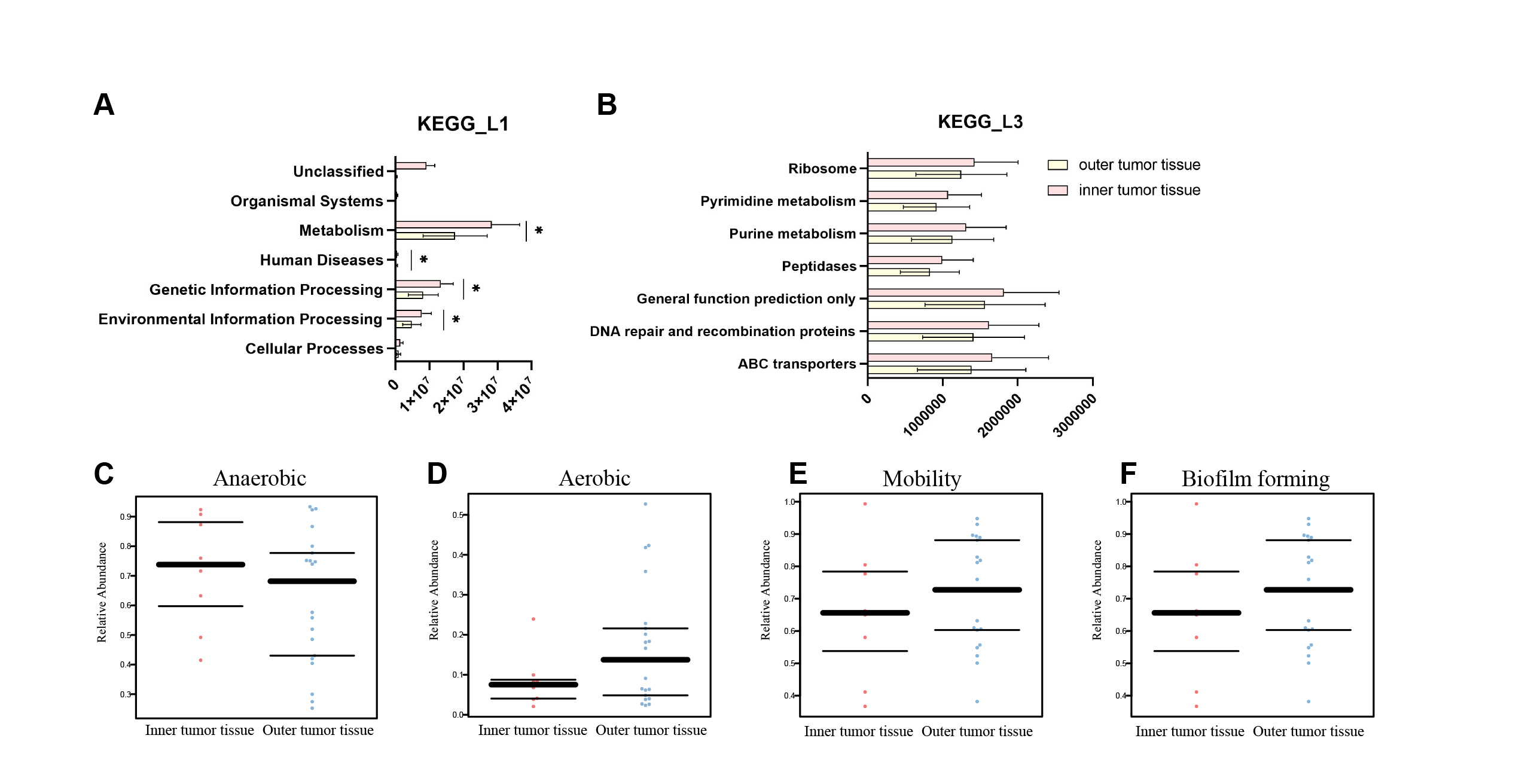

Supplement: Supplementary Figure 4 — (A, B) Bar plot of the predicted function of inner tumor tissues and outer tumor tissues at KEGG level 1 (A) and KEGG level 3 (B) by PICRUSt. (C–F) Scatter plot of predicted phenotypes of inner and outer tumor tissues by BugBase. Four kinds of phenotypes were shown: anaerobic (C), aerobic (D), mobility (E), and biofilm-forming (F). [file Image_4.jpeg]
