# Supplementary material for: The oral cancer microbiome contains tumor space–specific and clinicopathology-specific bacteria
Source: Front Cell Infect Microbiol. 2022 Dec 27;12:942328. doi: 10.3389/fcimb.2022.942328 (PMC9831678; doi:10.3389/fcimb.2022.942328)
Supplement: Supplementary Table 1 — Patient demographic and clinicopathological characteristics of the 27 patients in the microbiome analysis. [file Table_1.docx]

**Supplementary Table 1** Patient demographic and clinicopathological characteristics of 27 patients in the microbiome analysis

|  |  |  | Bacterial culture test | |  |
| --- | --- | --- | --- | --- | --- |
|  |  |  | Positive (N=11) | Negative (N=16) | *P* |
| Basic information | Age |  | 57.00±9.19 | 54.47±11.42 | 0.544 |
|  | Gender | Male | 8 | 10 | 0.692 |
|  |  | Female | 3 | 6 |  |
|  | Smoking | Yes | 6 | 8 | 0.816 |
|  |  | No | 5 | 8 |  |
|  | Drinking | Yes | 6 | 8 | 0.816 |
|  |  | No | 5 | 8 |  |
|  | Diabetes | Yes | 1 | 2 | 0.782 |
|  |  | No | 10 | 14 |  |
|  |  |  |  |  |  |
| Tumor features | Site |  |  |  | 0.743 |
|  |  | Tongue | 4 | 7 |  |
|  |  | Buccal | 2 | 2 |  |
|  |  | Gingival | 1 | 4 |  |
|  |  | Palate | 1 | 1 |  |
|  |  | Oral floor | 3 | 2 |  |
|  | Appearance classification |  |  |  | 0.144 |
|  |  | Exogenous | 5 | 6 |  |
|  |  | Ulcer | 4 | 10 |  |
|  |  | Infiltrating | 2 | 0 |  |
|  | Pathological T staging |  |  |  | **0.039*** |
|  |  | T1/T2 | 0 | 6 |  |
|  |  | T3 | 3 | 5 |  |
|  |  | T4 | 8 | 5 |  |
|  | Pathological N staging |  |  |  | 0.656 |
|  |  | N0 | 3 | 6 |  |
|  |  | N1 | 2 | 5 |  |
|  |  | N2 | 3 | 3 |  |
|  |  | N3 | 3 | 2 |  |
|  | Cervical lymph node metastasis | Yes | 8 | 10 | 0.692 |
|  |  | No | 3 | 6 |  |
|  | Pathological grading |  |  |  | 0.252 |
|  |  | High | 4 | 7 |  |
|  |  | Moderate | 7 | 9 |  |

Bold value with * indicates p value < 0.05.
